# Efficacy of the growing rod technique on kyphotic early-onset scoliosis

**DOI:** 10.3389/fped.2022.982295

**Published:** 2022-10-06

**Authors:** Jiang Cao, Xuejun Zhang, Jun Cao, Rongxuan Gao, Dong Guo

**Affiliations:** ^1^Guizhou Children's Hospital, Zunyi, China; ^2^Department of Pediatric Surgery, Affiliated Hospital of Zunyi Medical University, Zunyi, China; ^3^Department of Pediatric Orthopedics, Beijing Children's Hospital, Capital Medical University, Beijing, China

**Keywords:** kyphosis, early-onset scoliosis, growing rod technique, complication, pediatric surgery

## Abstract

**Objective:**

To explore the application of the growing rod (GR) technique in the treatment of kyphotic early-onset scoliosis (KEOS) and analyze its surgical efficacy and safety.

**Methods:**

The clinical data of 30 children with KEOS who received GR treatment at our department between January 2016 and December 2019 were analyzed retrospectively. There were 18 cases with normal kyphosis (normal kyphosis group) and 12 cases with excessive kyphosis (excessive kyphosis group). Both groups received GR treatment, and all patients received anteroposterior and lateral spine X-ray examinations before, after the initial surgery, and at the final follow-up. The surgical conditions and imaging parameters of the two groups were compared, and the complications were recorded.

**Results:**

There was no statistical difference in the Cobb angle of the major curve, apical vertebral translation (AVT), and trunk shift (TS) between the two groups before, after the first surgery, and at the final follow-up (*P* > 0.05). The Cobb angle of the major curve, the AVT, and the TS in both groups after the first surgery were lower than before the first surgery (*P* < 0.05), but there was no statistical difference between the two groups (*P* > 0.05). At the final follow-up, there were increases in both the Cobb angle and the AVT (*P* < 0.05), while the TS decreased in comparison with findings after the first surgery (*P* < 0.05). Before and after the first surgery and at the final follow-up appointment, there was a statistical difference in the degree of thoracic kyphosis (TK) between the two groups (*P* < 0.05), while there was no statistical difference in terms of lumbar lordosis (LL), the proximal junctional angle (PJA), and the distal junctional angle (DJA) (*P* > 0.05). After the first surgery, TK and LL showed a significant moderate response in both groups (*P* < 0.05), while there was no significant difference in TK, LL, PJA, and DJA compared with the results at the final follow-up (*P* > 0.05).

**Conclusions:**

The use of the GR technique can improve kyphosis in KEOS treatment.

## Introduction

Early-onset scoliosis (EOS), which is defined as scoliosis before the age of 10 years (1, 2), is a challenging condition in clinical practice, and the treatment for EOS has proved difficult in pediatric spinal surgery. Although early extensive fusion and fixation surgery can correct malformations, it affects the longitudinal growth of the spine and results in adverse consequences, such as short stature. More importantly, spinal growth restriction leads to the extrusion of the thorax and the lungs contained within, thereby impacting normal development and breathing (3). Therefore, to correct spinal malformation and preserve growth and lung development, non-fusion techniques have been applied clinically, among which, the growing rod (GR) technique is the most widely used. The objective of this surgical treatment is to partially correct malformation and prevent its progression. Meanwhile, the spine and thorax can continue to grow by regular extension, and orthopedic fusion fixation is performed until the bone is mature (4).

Currently, clinical efficacy assessment on EOS treatment using the GR technique is reflected mainly in the correction of coronal plane malformation. Research on the sagittal plane is limited, focusing mainly on sagittal plane improvement within the scope of instrument fixation, and there is a lack of research on the morphological changes of the spine outside the scope of instrument fixation (5).

Therefore, the present research aimed to study of a group of children with EOS who were treated with GRs and assess the morphological changes in and out of the range of sagittal plane instrument fixation to evaluate the effect of the GR technique on the sagittal plane of the spine.

## Materials and methods

### General information

The clinical data of 30 children (11 males and 19 females) with EOS who received GR treatment in our department between January 2016 and December 2019 were analyzed retrospectively. According to the reference value of T_2_-T_12_ for thoracic kyphosis (TK) in normal children, the 30 cases were divided into the following two groups: (1) The normal kyphosis group (*n* = 18) included 8 males and 10 females. They ranged in age from 4 to 9 years, and the average age was 6.1 ± 0.8 years. The preoperative TK was 20–50°, with an average of 34.6° ± 2.2°. (2) The excessive kyphosis group (*n* = 12) included 3 males and 9 females. The children ranged from 3 to 7 years of age, with an average of 5.3 ± 0.8 years. The preoperative TK was >50°, and the average was 67.5° ± 3.6°.

The inclusion criteria were as follows: (1) patients with EOS treated with the bilateral GR technique (age ≤ 10 years), (2) patients showing no response after correction with orthosis and plaster, (3) patients with initial lateral bending > 45° or continuous progression of lateral bending >10°/year, (4) patients who had received at least two strutting surgeries, and (5) patients receiving complete clinical imaging treatment with a follow-up time exceeding 2 years. The exclusion criteria were (1) patients whose cervical vertebrae were located in the upper thoracic vertebrae and the lumbar vertebrae, (2) patients exhibiting sagittal angular malformation, (3) patients whose lumbosacral vertebrae malformation had led to pelvis imbalance, (4) patients with a history of spinal surgery, and (5) patients suffering from malformation of the nervous system.

### Surgical method

**Figure 1:**
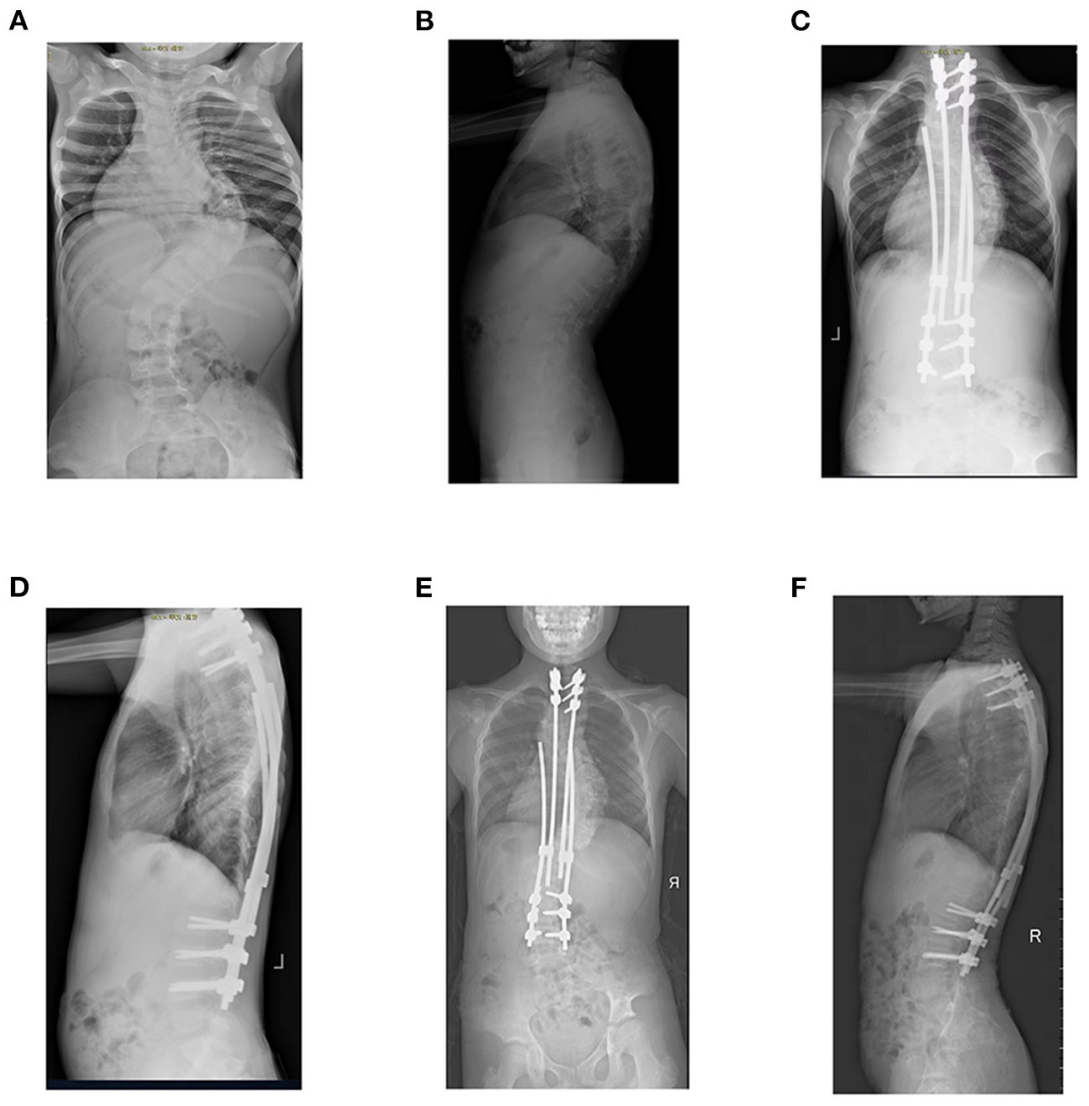
Boy aged 7 years and 8 months diagnosed with kyphotic early-onset scoliosis. **(A,B)** preoperative anteroposterior and lateral radiographs of the spine indicate coronal lateral bending of 91° and sagittal thoracic kyphosis of 63°. **(C,D)** X-ray re-examination shows satisfactory correction of kyphosis after growing-rod implantation. **(E,F)** X-ray re-examination at the 38th month of follow-up (the 4th strutting surgery) showed a satisfactory correction effect and an increase in spinal height.

The twin-rod technique was used to select proximal and distal anchor points according to the child's spinal malformation. The first surgery was performed under spinal cord monitoring, which included the application of somatosensory and motor-evoked potentials. The patient was placed in a prone position to determine the incision location and size. After the exposure of the proximal and distal anchor points, two or three pairs of pedicle screws were implanted into the proximal anchor point, and two pairs were implanted into the distal anchor point. Two titanium rods with a pre-formed sagittal curve were passed through the muscle surface below the deep fascia and connected to the proximal and distal anchor points and the domino connector, respectively. After appropriate strutting, both ends were locked. The reserved length of the connecting rod depended on the patient's growing potential. After the first surgery, strutting was performed every 7–9 months, during which a small cut was made each time at the domino connector for blunt dissection to the site to be strutted. Then, appropriate strutting instruments were used to extend the length of the connecting rod. The strutting length depended on the patient's growth and the severity of their spinal malformation (see Figure 1).

### Observation index

#### Surgical conditions

The surgical treatment conditions of both groups were compared, including the number of surgeries and the intervals between surgeries.

#### Imaging examination

The imaging information for both groups was compared. To measure the imaging parameters, lateral X-ray radiographs of the entire spine of all patients were taken in a standing position before, after the first surgery, and 2 years later, at the final follow-up.

The coronal plane parameters were as follows: ① Cobb angle of the major curve, i.e., the intersection angle between the tangent line of the upper endplate of the most slanted vertebral body and the tangent line of the lower endplate of the most slanted vertebral body on the anteroposterior X-ray radiograph of the entire spine in a standing position. ② Apical vertebral translation (AVT), i.e., the horizontal distance between the center of the apical vertebral body and the C7 vertebral body centered along a plumb line. ③ Trunk shift (TS), i.e., the distance between the vertical reference line of the trunk and the midperpendicular line of the sacrum.

The sagittal plane parameters were as follows: ① Thoracic kyphosis (T_2_-T_12_), i.e., the intersection angle between the tangent line of the upper endplate of T_2_ and the tangent line of the lower endplate of T_12_ on the lateral X-ray radiograph of the entire spine in a standing position. ② Lumbar lordosis (LL) (L_1_-S_1_), i.e., the intersection angle between the tangent line of the upper endplate of L_1_ and the tangent line of the upper endplate of S_1_ on the lateral X-ray radiograph of the entire spine in a standing position. ③ Proximal junctional angle (PJA), i.e., the intersection angle between the lower endplate of the fixed vertebra and the upper endplate of two vertebral bodies on the anteroposterior X-ray radiograph of the entire spine in a standing position. Proximal junctional kyphosis (PJK) is defined as PJA ≥ 10° and an increase of at least 10° for the same section compared with the pre-surgery value. ④ Distal junctional angle (DJA), i.e., the intersection angle between the upper endplate of the furthest fixed vertebra and the lower endplate of the first non-fixed vertebra. Distal junctional kyphosis (DJK) is defined as DJA ≥ 10° and an increase of at least 10° for the same section compared with the pre-surgery value.

#### Complications

The incidence of complications was compared between the two groups, and the perioperative complications of the two groups were recorded, including rod breakage, pedicle screw looseness, PJK, and DJK. Additionally, the incidence of various complications was calculated and summarized.

### Statistical analysis

This study used SPSS 20.0 (SPSS, U.S.A.) statistical software for data entry and the statistical analysis, and the measurement data were expressed in terms of mean and standard deviation (x ± s). A one-way analysis of variance was used to compare the imaging indices before surgery, after the first surgery, and at the final follow-up. The incidence of complications between the normal kyphosis group and the excessive kyphosis group was compared using Fisher's exact test. The significance level (α) was set at α < 0.05.

## Results

### Comparison of surgery number and interval between the two groups

In terms of the number of surgeries and the time intervals between strutting operations, there was no statistical significance between the normal kyphosis group and the excessive kyphosis group (*P* > 0.05), as shown in Table 1.

**Table 1:** Comparison of number and interval of surgeries between the two groups.

**Group**	**Number of surgeries (times)**		**Mean interval of strutting (month)**
	**Times of growing rod implantation**	**Number of strutting surgeries**	
Normal kyphosis group (18)	18	39	9.25 ± 1.60
Excessive kyphosis group (12)	12	24	8.93 ± 1.41
χ^2^/t	1.521		0.718
P	0.119		0.418

### Comparison of coronal plane indices on X-ray radiographs of the entire spine in a standing position between the two groups

There was no statistical difference in the Cobb angle of the major curve, the AVT, and the TS between the two groups before surgery, after the first surgery, and at the final follow-up (*P* > 0.05). In both groups, the Cobb angle of the major curve, the AVT, and the TS after the first surgery were lower than the pre-surgery values (*P* < 0.05), and there was no statistical difference between the two groups (*P* > 0.05). There was an increase in the Cobb angle and the AVT at the final follow-up (*P* < 0.05), while there was a decrease in the TS value (*P* < 0.05) compared with after the first surgery, as shown in Table 2.

**Table 2:** Comparison of coronal plane indexes on X-ray radiograph of the entire spine in standing position between the two groups ($\bar{x}$ ± *s*).

**Group**	**Cobb angle of major curve (** **°** **)**			**AVT (mm)**			**TS (mm)**		
	**Pre-operation**	**Post-operation**	**The last**	**Pre-operation**	**Post-operation**	**The last**	**Pre-operation**	**Post-operation**	**The last**
			**follow-up**			**follow-up**			**follow-up**
Normal kyphosis group	54.1 ± 2.3	27.9 ± 1.4^*^	30.4 ± 1.9^Δ^	40.3 ± 3.7	24.0 ± 1.8^*^	27.1 ± 1.9^Δ^	23.0 ± 2.4	14.2 ± 1.4^*^	10.9 ± 1.0^Δ^
Excessive kyphosis group	55.3 ± 2.5	26.1 ± 1.2^*^	29.7 ± 1.9^Δ^	39.2 ± 3.4	24.7 ± 1.8^*^	28.1 ± 1.9^Δ^	23.0 ± 2.7	14.0 ± 1.3^*^	10.4 ± 1.0^Δ^
t	1.285	1.593	0.431	0.859	1.228	1.350	0.021	0.398	1.470
*P*	0.209	0.123	0.669	0.398	0.230	0.188	0.983	0.694	0.153

* *P* < 0.05 compared with that before surgery;

Δ means *P* < 0.05 compared with that after surgery.

### Comparison of sagittal plane indices on X-ray radiographs of the entire spine in a standing position between the two groups

Before and after the first surgery and at the final follow-up, there was a statistical difference in TK between the two groups (*P* < 0.05), but there was no statistical difference in terms of LL, PJA, and DJA (*P* > 0.05). After the first surgery, there was a significant moderate response for both TK and LL in both groups (*P* < 0.05), while there was no significant difference in the TK, LL, PJA, and DJA values compared with those at the final follow-up (*P* > 0.05), as shown in Table 3.

**Table 3:** Comparison of sagittal plane indexes on X-ray radiograph of the entire spine in standing position between the two groups ($\bar{x}$ ± s).

**Group**	**TK(** **°** **)**			**LL(** **°** **)**			**PJA(** **°** **)**		**DJA(** **°** **)**	
	**Pre-operation**	**Post-operation**	**The last**	**Pre-operation**	**Post-operation**	**The last**	**Post-operation**	**The last**	**Post-operation**	**The last**
			**follow-up**			**follow-up**		**follow-up**		**follow-up**
Normal kyphosis group	34.6 ± 2.2	28.1 ± 1.4^*^	30.5 ± 1.7^Δ^	42.6 ± 2.6	36.0 ± 1.4^*^	38.2 ± 2.0^Δ^	12.1 ± 1.4	14.0 ± 1.9^Δ^	11.2 ± 1.4	11.9 ± 1.8^Δ^
Excessive kyphosis group	67.5 ± 3.6	47.4 ± 1.3^*^	49.7 ± 1.8^Δ^	49.3 ± 2.6	40.8 ± 1.4^*^	40.4 ± 2.0^Δ^	12.2 ± 1.4	13.8 ± 1.9^Δ^	11.1 ± 1.3	12.5 ± 1.8^Δ^
t	0.283	0.305	0.340	0.711	1.398	0.244	0.132	0.200	0.281	0.532
*P*	0.015	0.002	0.031	0.483	0.173	0.809	0.896	0.843	0.781	0.599

* *P* < 0.05 compared with that before the first surgery;

Δ means *P* > 0.05 compared with that after the first surgery.

### Comparison of incidence of complications between the two groups

Until the final follow-up, the incidence of pedicle screw looseness in the normal kyphosis group was lower than in the excessive kyphosis group (*P* < 0.05), and there was no statistical difference in terms of rod breakage, PJK, and DJK (*P* > 0.05) between the groups, as shown in Table 4.

**Table 4:** Comparison of incidence of complications between the two groups [*n* (%)].

**Group**	**Rod breaking**	**Pedicle screw loosening**	**PJK**	**DJK**
Normal kyphosis group	2 (11.11)	1 (5.56)	2 (11.11)	1 (5.56)
Excessive kyphosis group	4 (33.33)	4 (33.33)	3 (25.00)	2 (16.67)
χ^2^	2.222	4.000	1.000	0.988
*P*	0.136	0.046	0.317	0.320

## Discussion

Early-onset scoliosis occurs at a young age. The younger the child, the greater the growth potential of the spine. First, the initial peak of spinal growth and development occurs between 0 and 5 years, so there is a high risk of scoliosis occurring and progressing rapidly. Second, the young age of patients leads to poor compliance and non-cooperation in the treatment process, especially when receiving conventional treatment. Thus, the efficacy of treatment is often poor (1). Third, the cardiopulmonary development of children with EOS is immature, and severe thoracic scoliosis may lead to cardiopulmonary dysfunction. For patients requiring surgical treatment, their organ functions, including cardiopulmonary function, are not fully developed, and their tolerance is low. All these factors increase surgical risk (6). Consequently, EOS often requires a long treatment cycle that demands patience from the parents of those being treated. Accordingly, EOS poses a major challenge for both doctors and families.

The conservative treatment efficacy for children with EOS is poor, and conventional fusion and fixation surgery results in a short trunk, a disproportionate body shape, and the crankshaft phenomenon. The GR technique is a common non-fusion operation method. It mainly controls the progression of scoliosis by determining the upper and lower anchor points of the spine and implanting the GR to strut the spine regularly. Meanwhile, the premature fusion of the vertebrae is prevented by the application of a longitudinal growing force and the provision of sufficient growth space for the spine and thoracic vertebrae (7). The results of the present study showed that until the final follow-up, the number of surgeries and to the time between procedures were ideal for all children, indicating that the GR technique had a good surgical effect. No significant differences were observed between the normal kyphosis group and the excessive kyphosis group.

Early-onset scoliosis is characterized by early onset and rapid progression and requires early surgical intervention. For short-segment malformation without compensatory curvature or for mild compensatory malformation, good clinical efficacy has been demonstrated with the short-segment fusion of hemivertebra resection or total spinal resection (8). However, EOS involving a large range of spinal structural disorders (e.g., multiple hemivertebrae, butterfly vertebrae, and wedge vertebrae in the lateral curvature) has posed problems in spinal malformation treatment (9). In 2012, Zhang et al. (10) reported the clinical effect of the double GR technique in the treatment of 30 children with scoliosis: 72.30 before scoliosis surgery, 34.90 after the first surgery, and 36.20 at the final follow-up. The annual growth rate of T_1_-S_1_ was 1.49 cm/year, and the SAL was 0.81, 0.95, and 0.96, respectively, before surgery, after the first surgery, and at the final follow-up. Accordingly, it can be seen that the GR technique has good efficacy for EOS.

The results of this study showed that after the first GR surgery for long-segment EOS, the coronal and sagittal plane parameter indices were clearly corrected. In the normal kyphosis group, the Cobb angle of the major curve on the coronal plane decreased to 27.9° ± 1.4° after surgery. There were post-surgery decreases in the AVT (to 24.0 ± 1.7 mm), the TS (to 14.2 ± 1.4 mm), the TK on the sagittal plane (to 28.1° ± 1.4°), and the LL (to 36.0° ± 1.4°). The PJA and DJA were 14.0° ± 1.9° and 11.9° ± 1.4°, respectively, after surgery, and there was only a slight increase in the long-term follow-up, although there was no statistical difference (*P* > 0.05). Thus, it is preliminarily believed that the GR technique is a satisfactory choice for EOS with good flexibility and without other malformations (11, 12). The initial surgery effectively improved spinal malformation, and the orthopedic effect was satisfactory after regular strutting until the final follow-up. After further analyses of the coronal plane and sagittal plane parameters in the normal kyphosis and the excessive kyphosis groups, statistical differences were found in the TK values between the two groups before surgery, after the first surgery, and at the final follow-up (*P* < 0.05); additionally, there was no significant difference in the other parameters at each time point (*P* > 0.05). These results confirm that the coronal malformation of scoliosis had a significant moderate response after treatment with the GR technique. Although TK in the excessive kyphosis group was greater than in the normal kyphosis group before surgery, after the first surgery, and at the final follow-up, the degree of TK in the excessive kyphosis group decreased from 67.5° ± 3.6° before the first surgery to 47.4° ± 1.3° afterward, with a result of 49.3° ± 2.6° at the final follow-up, indicating that the double GR technique had a certain correction effect on sagittal malformation; however, the method could not reduce the degree of TK in the excessive kyphosis group closer to that in the normal kyphosis group in terms of sagittal malformation. Previously, it was believed that the GR technique had a limited effect on the correction of sagittal malformation and, to prevent complications, it should not be used to correct sagittal kyphosis. However, as experience with the treatment increased, some scholars reported the correction effect of the GR technique on sagittal malformation. For instance, Chen et al. (13) reported that the GR technique could effectively reconstruct sagittal balance in kyphotic EOS. Han et al. (14) reported that as a “good” treatment method, the GR technique had a positive effect on the recovery of the full sagittal spinal sequence. The present study is essentially consistent with the conclusions reported in the literature.

In terms of complications, after treatment with the GR technique, the main complications included rod breakage, pedicle screw looseness, PJK, and DJK. On the whole, the number of complications was low. However, due to the low number of cases, the incidence of complications was high. If the number of cases increased to a certain level, the incidence of complications would decrease significantly. Thus, the GR technique is relatively safe in the treatment of EOS (13). In terms of complications in the normal kyphosis and the excessive kyphosis groups, there were certain differences in the incidence of pedicle screw looseness (*P* < 0.05). The structural characteristics of the GR technique mean that the stress of internal fixation, especially at the proximal anchor point, is high during the strutting treatment process, easily resulting in complications related to internal fixation. Yao et al. (15) reported that the incidence of complications related to internal fixation in patients with EOS reached 28.8% (*n* = 17/59); they considered the risk factors to be an age of < 9 years, TK > 50°, and the use of the GR technique.

Therefore, in the present study, the incidence of complications in the excessive kyphosis group was higher than in the normal kyphosis group. Accordingly, before treatment, the malformation of patients with EOS must be assessed fully, and attention must be paid to sagittal malformation. All children included in this study were treated with the double GR technique. To minimize complications and improve the safety of GR treatment, the double rods can be pre-bent for TK and LL and inserted in the muscles below the deep fascia. Meanwhile, the domino connector is placed under the deep fascia of the thoracolumbar segment, where it is opened regularly. To further reduce complications, for the children with EOS in the excessive kyphosis group, three fixation segments should be selected for the proximal anchor point, and pedicle screws should be used. If screw placement is difficult, the lamina hook or the transverse process hook may be used instead to increase the stability of the proximal internal fixation. If patients have stiff and severe kyphosis, osteotomy may be performed in the apical vertebra, and the correction rate of scoliosis and kyphosis malformation may increase on the initial surgery side, thus reducing the incidence of complications. If necessary, halo head-ring gravity traction may be used before GR implantation surgery to increase the flexibility of the scoliosis.

However, this study has some limitations, such as its small sample size, which may have affected the results. Furthermore, the follow-up time needs to be extended further. In the future, larger comparative case series studies will be continued.

In conclusion, the GR technique has a significant effect on the treatment of children with EOS in terms of improving spinal coronal and sagittal parameters, contributing to the recovery of spinal function, and ensuring surgical safety. However, it is necessary to prevent complications, such as rod breakage and pedicle screw looseness.

## Data availability statement

The original contributions presented in the study are included in the article/supplementary material, further inquiries can be directed to the corresponding author/s.

## Ethics statement

The studies involving human participants were reviewed and approved by Ethics Committee of Beijing Children's Hospital. Written informed consent to participate in this study was provided by the participants' legal guardian/next of kin.

## Author contributions

XZ: conception and design of the research. JuC and RG: acquisition of data, analysis and interpretation of the data, and statistical analysis. JiC: obtaining financing. DG and JiC: writing of the manuscript. DG: critical revision of the manuscript for intellectual content. All authors read and approved the final draft.

## Funding

This work was supported by Department of Science and Technology of Guizhou Province, Project name: High-level Innovative Talents of Guizhou Province ([2020]6015).

## Conflict of interest

The authors declare that the research was conducted in the absence of any commercial or financial relationships that could be construed as a potential conflict of interest. Handling editor PY is at the same institution as authors XZ, JuC, RG, and DG.

## Publisher's note

All claims expressed in this article are solely those of the authors and do not necessarily represent those of their affiliated organizations, or those of the publisher, the editors and the reviewers. Any product that may be evaluated in this article, or claim that may be made by its manufacturer, is not guaranteed or endorsed by the publisher.
